# Success factors and measures for scaling patient-facing digital health technologies from leaders’ insights

**DOI:** 10.1186/s12913-025-12748-z

**Published:** 2025-05-01

**Authors:** Estelle Pfitzer, Odile Giger, Christoph Kausch, Tobias Kowatsch

**Affiliations:** 1 Centre for Digital Health Interventions, School of Medicine, University of St. Gallen, St. Gallen, Switzerland; 2 MTIP AG, Basel, Switzerland; 3 Centre for Digital Health Interventions, Institute for Implementation Science in Health Care, University of Zurich, Zurich, Switzerland; 4 Centre for Digital Health Interventions, Department of Management, Technology, and Economics, ETH Zurich, Zurich, Switzerland

**Keywords:** Digital health technologies, Patient-facing solutions, Success factors, Scaling strategies, Non-communicable diseases

## Abstract

**Background:**

Europe’s healthcare systems face a triple burden: the rise of non-communicable diseases (NCDs), an aging population, and a shortage of healthcare professionals. NCDs, the leading causes of death, disproportionately affect older adults, placing significant pressure on healthcare services. By 2050, nearly 30% of Europe’s population will be aged 65 or older, up from 20% in 2023. These challenges demand urgent solutions to sustain healthcare systems. Patient-facing digital health technologies (DHTs), such as Digital Diagnostics and Digital Therapeutics, offer promising tools to address this burden by empowering patient self-management, reducing strain on healthcare professionals, and enhancing system efficiency. Despite their potential, the scaling and adoption of DHTs remain limited. This study investigates: (RQ1) What key factors drive success across different patient-facing DHT categories? and (RQ2) How can companies implement these factors?

**Methods:**

Following COREQ guidelines, we conducted semi-structured interviews with 29 executives and founders of European DHT companies targeting NCDs. Participants were identified using PitchBook, focusing on revenue-generating companies with over 20 employees. Virtual interviews were conducted in English between May and September 2024, lasting an average of 28 min (range: 21–40). Data saturation determined the sample size. Thematic analysis was performed, with two researchers independently coding the data to ensure reliability. Success factors were categorized as internal (e.g., employees) or external (e.g., partnerships). Ethical approval was obtained, and data was anonymized. A follow-up survey (*n* = 27) was conducted to confirm our findings.

**Results:**

We identified 18 success factors for scaling patient-facing DHTs. Health & Wellness companies prioritized business model flexibility, while Digital Therapeutics relied on regulatory compliance. Validation of health impact was critical across categories, emphasized by all respondents in Digital Diagnostics and Digital Therapeutics. Other key factors included customer awareness, strategic partnerships, and investor alignment, highlighting the importance of tailored growth strategies.

**Conclusion:**

This study provides structured guidance for scaling patient-facing DHTs, emphasizing category-specific strategies aligned with operational, regulatory, and consumer demands. It offers actionable recommendations for founders and executives to allocate resources effectively and adapt to diverse market contexts. By addressing the unique challenges of scaling DHTs, this work contributes to advancing digital health research and improving healthcare system resilience.

**Supplementary Information:**

The online version contains supplementary material available at 10.1186/s12913-025-12748-z.

## Background

European healthcare systems stand at a critical crossroads, driven by the escalating costs of non-communicable diseases (NCDs) and the rapid aging of the population [1–3]. By 2050, nearly one-third of individuals will be aged 65 or older, those most vulnerable to NCDs, compared to one-fifth in 2023 [4]. This comes with a growing shortage of healthcare professionals in Europe, described by the WHO as a “ticking time bomb,” warning that without immediate action, these workforce gaps could have catastrophic consequences [5–7]. Patient-facing digital health technologies (DHTs), as defined by the DTx Alliance, include products and services with patient-facing features across the categories of Health and Wellness, Care Support, Patient Monitoring, Digital Diagnosis, and Digital Therapeutics [8]. These technologies offer a promising solution to the growing mismatch between healthcare service supply and demand by empowering patients to take a more active role in managing their health [9–11] and improving the efficiency of healthcare workers [12, 13]. By addressing these critical challenges, DHTs can help sustain the quality of care in an increasingly strained system and contribute to the healthcare triple aim: improving the experience of care, enhancing population health, and reducing per capita healthcare costs [14].

Despite substantial investments and the increasing number of DHTs in recent years, their adoption has been slow, and only a few have been effectively used and scaled in Europe [15–19]. A notable example of slow digital health adoption is Germany’s Digital Health Applications (DiGA) program, which enables certified digital health tools to be prescribed and reimbursed through statutory health insurance [20]. In spite of 70% of doctors being aware of DiGAs, only 7% actively prescribe them [21]. Transitioning from provisional to permanent listing can take up to two years, highlighting the challenge of generating robust, long-term clinical evidence to prove efficacy [22]. Initially, manufacturers are free to set the price for their applications, but following negotiations with regulatory bodies, the average price is typically reduced by more than 50%. This significant price drop can hinder company growth and complicate the economic viability of DHTs within the program​ [23].

In spite of the challenges in the DHT environment, some companies have managed to scale successfully. For example, Epic Systems, a leading electronic health record provider, was rated as the best overall electronic health record suite for the 13th consecutive year in 2023 and is currently in use in 89% of acute care hospitals in the United States [24], playing an important role in supporting healthcare service delivery. However, many digital health companies have struggled to achieve sustainable growth despite healthcare systems’ evident need to adopt such technologies [25]. It is, therefore, important to understand what hinders or, most importantly, unlocks their widespread adoption.

### Knowledge gap

While some barriers to DHTs’ integration in healthcare have been researched [26–32], a comprehensive and structured analysis of best practices and strategies that contribute to the success of patient-facing European digital health companies remains absent, to our knowledge. Specifically, our literature review [33] on the success factors of growth-stage digital health companies revealed that each DHT category, as defined by the DTx Alliance [34], should prioritize distinct success factors. However, there is scarce research guiding these priorities. This gap is also evident in WHO’s Digital Health guidelines, where recommendations for implementing DHTs are made while acknowledging there is limited or no evidence to support their effectiveness in successfully scaling digital health companies [35]– a crucial step to achieving widespread adoption and addressing pressing healthcare challenges, such as improving patient outcomes and alleviating the healthcare workforce shortage.

### Objectives of the study

Our study builds upon our systematic literature review [33] and addresses the knowledge gap by providing DHT category-specific success factors and recommendations for scaling digital health companies. This knowledge fills the research gap and empowers digital health stakeholders to best position DHT companies for widespread adoption and growth. We tapped into the knowledge of executives and founders within the field to answer the following research questions:

- RQ1. What key factors contribute to companies’ success across different patient-facing DHT categories?
- RQ2. How do patient-facing DHT companies implement key factors contributing to their success?

## Methods

### Design and settings

A qualitative approach with semi-structured interviews was chosen to capture the in-depth lived experiences of executives and founders of patient-facing DHT companies across Europe [36, 37].

Europe was selected due to its diverse healthcare regulatory landscapes, languages, and varying levels of digital health adoption, which present unique challenges for DHT companies [30, 38, 39]. We conducted interviews virtually to ensure participation from a geographically diverse group of executives and founders, minimizing logistical constraints and accommodating their tight schedules.

We specifically targeted companies addressing NCDs, as these chronic conditions are responsible for most deaths and impose a high-cost burden on healthcare systems in Europe [40, 41]. DHTs can significantly impact this area by enabling prevention, early intervention, and long-term management and treatment of chronic diseases [42–44].

The interview study was designed and reported following the Consolidated Criteria for Reporting Qualitative Research (COREQ) guidelines to ensure methodological rigor (Supplementary Material 1) [45].

### Participant selection

We aimed to interview executives and founders from leading European DHT companies. To identify suitable companies, we used PitchBook, a database commonly used in related work [46–48]. Our inclusion criteria focused on Europe-headquartered companies within the Digital Health sector that are generating revenue. Additionally, we filtered for companies with over 20 full-time employees (FTEs) specializing in DHTs targeting NCDs. Details of the exact search strategy can be found in Supplementary Material 2.

We exported a structured list of companies from PitchBook, ranking companies by employee count in descending order to prioritize larger and more established firms. We then reached out to executives and founders, starting with the organizations with the most FTEs, to maximize insights from those leading sizable operations and progressing through the list to ensure diversity in company size and expertise.

### Data collection

Interviews were conducted in English from May to September 2024 until data saturation was achieved. Data saturation occurs when no new information or themes emerge from subsequent interviews [37, 49]. At the start of each interview, participants were given a brief overview of the interview study and its objectives. They were then asked to reflect on success factors contributing to their company’s current growth stage.

We explored each identified success factor in detail, focusing on the challenges encountered and strategies implemented to achieve these outcomes. Following this, we investigated participants’ perspectives on how these success factors will likely evolve over the next 5–10 years, aiming to uncover future strategic adaptations.

The topic guide for the interviews was informed by our systematic literature review [33] to establish a theoretical foundation (Supplementary Material 3). Interviews lasted, on average, 28 min and ranged between 21 and 40 min, and all were audio-recorded with the participant’s consent. Transcriptions were performed using Firefly.ai software, followed by a review process to ensure accuracy and fidelity.

### Data analysis and synthesis

All interview transcripts were imported into ATLAS.ti 24.2.0 [50] for qualitative coding and analysis. Two independent researchers (EP and OG) conducted an initial review to ensure reliability and reduce bias. The analysis proceeded in two phases: initially, a deductive thematic analysis was performed to establish an initial codebook based on our literature review [33]. This codebook was iteratively refined through multiple discussion rounds, consolidating and organizing the codes into a comprehensive resource (Supplementary Material 4).

Codes within the codebook were grouped into higher-order constructs, organizing success factors into broader themes referred to as segments. To ensure that our analysis reflected commonly agreed-upon factors, only those mentioned by at least five different interviewees were considered significant enough to be included. The initial codes were refined through iterative discussions, with some combined or removed as needed. Based on our prior work [33], success factors were classified as internal factors if they were within a company’s control (e.g., Employees or Business Model) or external factors if they were influenced by external stakeholders (e.g., Partnerships, Healthcare System).

In the second phase, an inductive approach was used to document the specific measures and capabilities critical for patient-facing DHT companies to achieve success factors. Finally, a third researcher reviewed the completed analysis for consistency and accuracy, ensuring that codes and themes were captured and represented accurately.

Finally, to further validate our findings, we performed a member-checking step by sending the manuscript and the finalized list of success factors to all participants. Any feedback received during this process was integrated into the final analysis, and in the absence of a response within a month, we considered the findings as confirmed.

### Follow up-survey

To validate and assess the relative importance of the measures identified in the interviews, we conducted a follow-up survey using Qualtrics (version February 2025) [51] targeting the same group of executives and founders we originally reached out to for interviews. This included both those who participated in the qualitative study and those who had been contacted but did not take part in an interview. The survey aimed to quantify the perceived significance of each measure in achieving the success factors identified through qualitative analysis. Respondents were asked to evaluate the relevance of specific measures associated with success factors, enabling a structured assessment of their relative weight and prioritization. The survey questions can be found in Supplementary Material 6.

## Results

### Characteristics of participants and companies

We conducted 29 interviews, with each participant representing a different company. All interviews were included in the analysis. The participants and their company characteristics are summarized in Table 1. Most participants held the Chief Executive Officer (CEO) position. Additionally, we interviewed an ex-Chief Technical Officer (CTO) of a recently acquired company and a Chief Operating Officer (COO). Of the 29 participants, 27 were male, and two were female.

**Table 1:** Demographic and company characteristics of interview participants

Participant characteristics		Participants
**Role, *n*(%)**		
CEO		27 (93%)
COO		1 (3%)
CTO		1 (3%)
**Gender, *n* (%)**		
Female		2 (7%)
Male		27 (93%)
**Company Headquarter, *n* (%)**		
Austria		1 (3%)
Czech Republic		1 (3%)
Denmark		3 (10%)
Finland		4 (14%)
France		1 (3%)
Germany		1 (3%)
Italy		1 (3%)
Netherlands		3 (10%)
Poland		2 (7%)
Sweden		2 (7%)
Switzerland		1 (3%)
Ukraine		1 (3%)
United Kingdom		8 (28%)
**Digital Health Technology category, *n* (%)**		
Care Support		6 (21%)
Digital Diagnostics		5 (17%)
Digital Therapeutics		7 (24%)
Health and Wellness		5 (17%)
Patient Monitoring		6 (21%)
**Disease area, *n* (%)**		
Cancer		4 (14%)
Cardiovascular Disease		1 (3%)
Cronic Respiratory Disease		4 (14%)
Diabetes		3 (10%)
Disease Agnostic		11 (38%)
Mental Health		6 (21%)
**Number of Employees, *n* (%)**		
20–50		16 (55%)
50–100		5 (17%)
100–200		4 (14%)
200+		4 (14%)

Geographically, we interviewed participants from a range of regions across Europe. In the Nordics, companies were headquartered in Finland (*n* = 4) and Sweden (*n* = 2). One company was based in France, and another in Italy. The DACH region (Austria *n* = 1, Germany *n* = 1, and Switzerland *n* = 1) was also represented, alongside Eastern Europe, with participants from Poland (*n* = 2), Ukraine (*n* = 1), and the Czech Republic (*n* = 1). The country with the most interviewees was the United Kingdom (*n* = 8), and we also interviewed three participants from the Netherlands (*n* = 3).

We achieved comprehensive coverage across patient-facing DHT categories, with five to seven interviews in each of the five key areas: Health & Wellness (*n* = 5), Patient Monitoring (*n* = 6), Care Support (*n* = 6), Digital Diagnostics (*n* = 5), and Digital Therapeutics (*n* = 7) [34].

Most companies our interviewees worked for had between 20-50 FTEs. Five companies had 50–100 FTEs, and four had 100–200 FTEs. Additionally, we interviewed four CEOs of companies with over 200 FTEs, some of which had more than 500 employees.

### Success factors

Our study set out to identify and map the essential success factors within each DHT category. Despite conducting a comprehensive systematic literature review, we encountered notable limitations due to insufficient research on several key categories, namely, Digital Therapeutics, Digital Diagnostics, and Health & Wellness, where little to no published studies addressed the critical factors needed for effective scaling. Building on *that* foundation, *this* interview study enabled us to refine our initial list down to the 18 most significant success factors and determine the relative importance of both internal and external factors within each DHT category. These 18 factors, together with their descriptions, the number of interviews in which they were cited, and the average FTE count of the companies that mentioned them, are presented in Table 2.

**Table 2:** Key success factors for patient-facing DHTs. “Mentions” indicates the total number of interviews in which each factor was cited, while “average FTE number” represents the average number of full-time employees in the companies that mentioned this factor. “I” denotes internal success factors, and “E” denotes external success factors

Segment	Factor	Description	Mentions	Average FTE number
I1. Business Model	a. Business model flexibility	The ability to adjust and experiment with different business models based on market feedback and evolving conditions	11	77
	b. Internationalization strategy	Expanding into new geographical markets, taking into account local regulations, culture, and market needs	9	111
	c. Market positioning strategy	Deliberate choice of where and how a solution is introduced, such as in consumer, less-regulated arenas or directly within clinical settings	7	169
	d. Sales and marketing strategy	Strategically placing the company or product in the market to differentiate it from competitors and maximize its appeal	8	119
I2. Product & Service	a. Product-market fit	Ensuring that there is a clear demand for the product and that it addresses significant customer needs	10	65
	b. Regulatory certification	Achieving necessary approvals and certifications from regulatory bodies to legally market products, especially in healthcare	11	57
	c. Quality and performance	Delivering consistently high-quality products that build trust and strengthen the brand’s reputation	5	47
I3. Employees	a. Leadership experience	Strong leadership that drives the team towards achieving the company’s vision through experience, motivation, and decision-making	7	122
	b. Diversity of expertise within employees	Having a team with varied skills, backgrounds, and perspectives to drive innovation and tackle complex problems	7	63
	c. Employee alignment with the company’s vision	A strong, clear company purpose that motivates employees and aligns them with the broader goals of the business	6	124
E1. Healthcare System	a. Health impact proof and validation	Offering solutions that enhance medical diagnoses or improve patient care outcomes	18	81
	b. Regulatory environment and policy framework	Navigating the legal and regulatory landscape to ensure compliance with evolving laws and standards	14	64
	c. Financial impact proof and validation	Solutions that help lower healthcare costs for providers, insurers, or patients, making care more efficient and affordable	9	50
E2. Customers	a. Customer feedback	Regularly gathering and acting on customer insights to improve product performance and customer satisfaction	13	106
	b. Customers awareness raising	Efforts to inform and educate potential customers about the product, its benefits, and its relevance to their needs	10	122
	c. Regional market size and customer behaviour	Evaluating demand, cultural factors, and openness to digital solutions in different regions to guide market expansion strategies	5	41
E3. Partnerships	a. Collaboration with larger organizations	Strategic partnerships with larger firms to access new markets, share resources, or enhance credibility	12	134
	b. Investor backing and fit	Aligning with investors whose vision, goals, and resources support the company’s long-term strategy and growth	8	134

#### Internal success factors

**Fig. 1:**
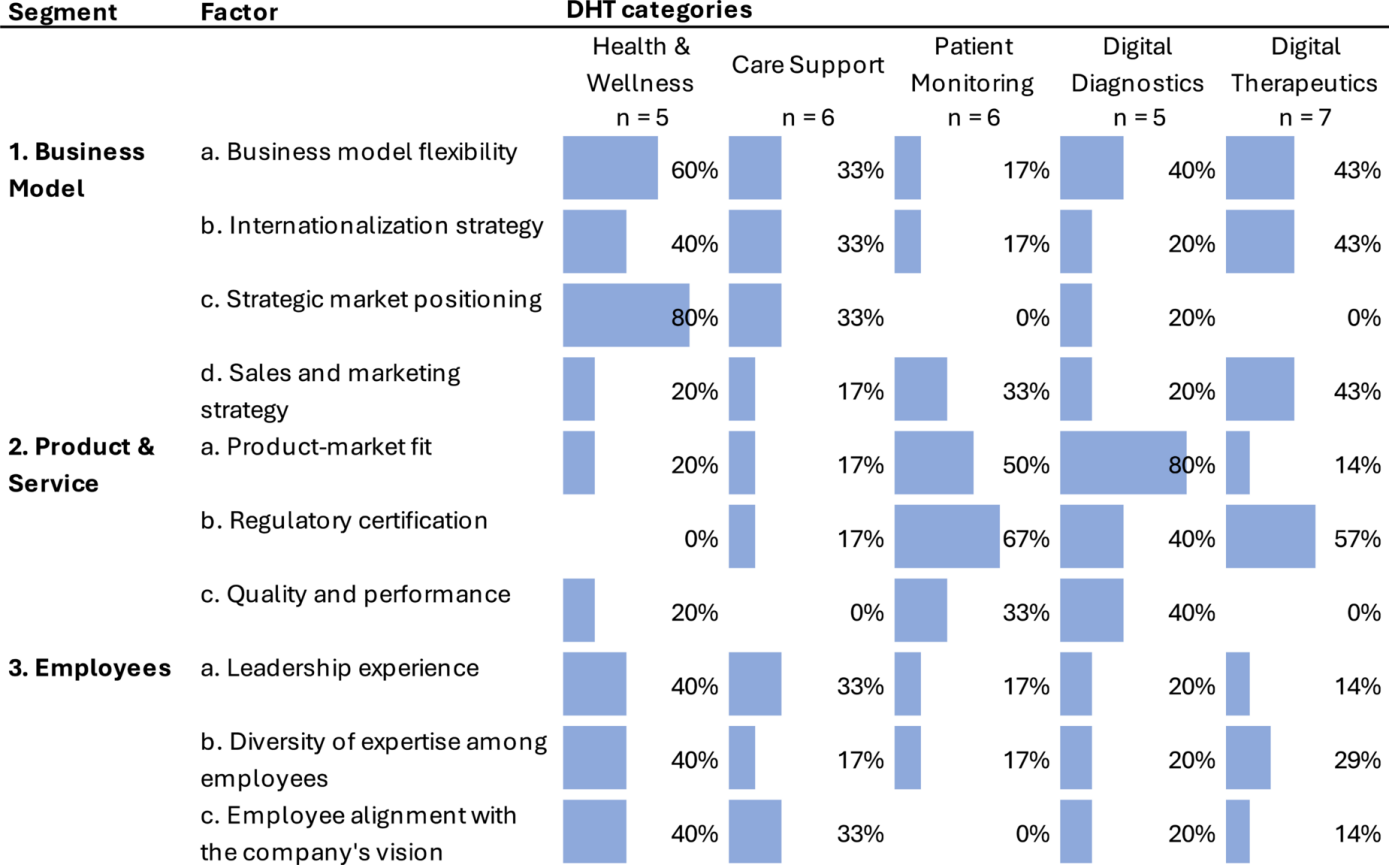
Internal success factors of patient-facing DHT companies. The total number of interviews per category is indicated beneath each category title, and the percentage and corresponding bar plot represent the frequency of each success factor. The DHT categories are listed in order of increasing regulatory intensity

Our interviews revealed ten key internal factors crucial for patient-facing DHT companies to achieve operational and financial success. Findings highlighted the diversity of success factors across patient-facing DHT categories (Fig. 1), emphasizing that a universal scaling strategy does not exist; instead, each category benefits from distinct approaches.

For Health & Wellness companies, Business Model factors were predominant. The most critical was a clear *strategic market positioning* (*n* = 4/5, 80%), followed by *business model flexibility* (*n* = 3/5, 60%) and a well-defined *internationalization strategy* (*n* = 2/5, 40%). Employee-related factors also played a significant role in this category, with attributes like *leadership experience*, *diversity of expertise*, and *alignment with the company’s vision* each highlighted (*n* = 2/5, 40%).

Care Support companies showed a pattern similar to Health & Wellness companies, prioritizing *Business Models* and *Employee-*related *factors*.

In the Patient Monitoring category, *Product and Service-related factors* were most critical. Here, *regulatory certification* emerged as the top priority (*n* = 4/6, 67%), followed closely by *product-market fit* (*n* = 3/6, 50%). Additional influential factors included the *quality and performance* (*n* = 2/6, 33%) of products or services and a strong *sales and marketing strategy* (*n* = 2/6, 33%).

For Digital Diagnostics companies, *product-market fit* in the Product & Service segment was reported as the most crucial (*n* = 4/5, 80%). Other essential factors highlighted by CEOs and founders included *regulatory certification* (*n* = 2/5, 40%) and *quality and performance* (*n* = 2/5, 40%).

In the Digital Therapeutics category, *regulatory certification* emerged as the single most important success factor (*n* = 4/7, 57%). Business model factors were also significant, with factors like *business model flexibility* (*n* = 3/7, 43%), *internationalization strategy* (*n* = 3/7, 43%), and *sales and marketing strategy* (*n* = 3/7, 43%) each cited by nearly half of the participants.

#### External success factors

**Fig. 2:**
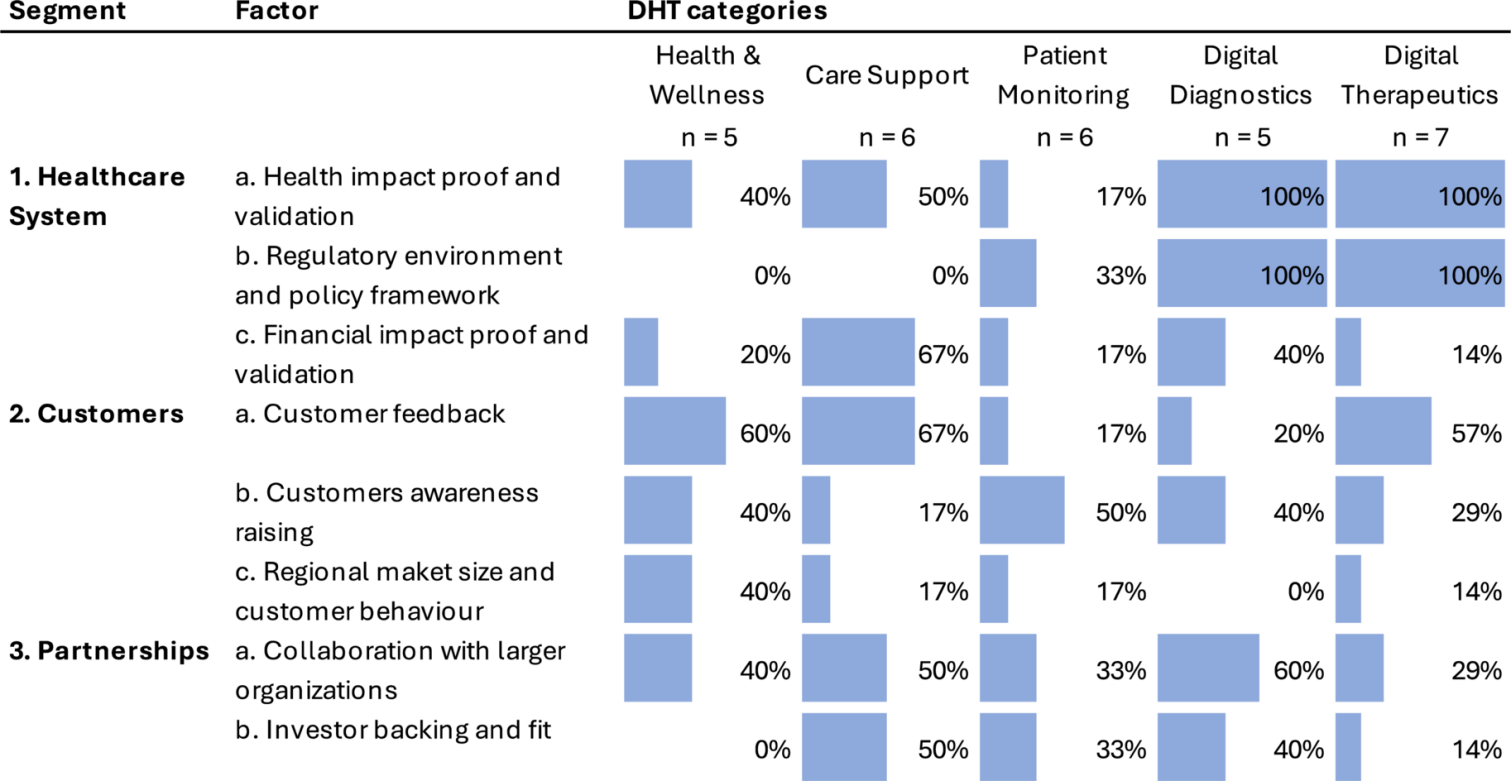
External success factors of patient-facing DHT companies. The total number of interviews per category is indicated beneath each category title, and the percentage and corresponding bar plot represents each success factor’s frequency. The DHT categories are listed in order of increasing regulatory intensity

Our semi-structured interviews revealed eight key external success factors related to stakeholders in the Healthcare System, Customers, and various Partnerships (Fig. 2).

For Health & Wellness companies, Customer success factors were the most frequently reported. Gathering *customer feedback* (*n* = 3/5, 60%), *raising customer awareness* (*n* = 2/5, 40%), and understanding the *regional market size and customer behavior* (*n* = 2/5, 40%) were each highlighted by two or more founders and CEOs in digital Health & Wellness. Although the *regulatory environment and policy framework* (*n* = 0/5, 0%) were not discussed, demonstrating *health impact proof and validation* (*n* = 2/5, 40%) remains critical for large-scale adoption.

In contrast, Care Support companies prioritize *financial impact proof and validation* (*n* = 4/6, 67%) and *health outcomes proof and validation* (*n* = 3/6, 50%) to facilitate integration within healthcare systems. *Customer feedback* (*n* = 4/6, 67%) was also regarded as important. Additionally, *collaboration with larger organizations* (*n* = 3/6, 50%) and securing the right *investor backing and fit* (*n* = 3/6, 50%) were viewed as essential partnerships for scaling.

In the Patient Monitoring category, companies should primarily focus on *raising customer awareness* (*n* = 3/6, 50%) and securing *key partnerships* with larger organizations (*n* = 2/6, 33%). Additionally, *finding the right investor backing* (*n* = 2/6, 33%) is essential for sustained growth. As regulatory demands increase, the importance of the *regulatory environment and policy framework* (*n* = 2/6, 33%) is also increasingly recognized.

In the Digital Therapeutics category, establishing *health impact proof and validation* (*n* = 6/7, 86%) was seen as the most critical factor for scaling, alongside ensuring compliance with the *regulatory environment and policy framework* (*n* = 5/7, 71%). *Financial impact proof and validation* (*n* = 4/7, 57%) were also raised by CEOs and founders in this category. Partnerships were similarly emphasized, with *collaboration with larger organizations* (*n* = 3/7, 43%) and *investor backing and fit* (*n* = 3/7, 43%) highlighted. *Customer awareness raising* was reported as beneficial (*n* = 3/7, 43%) to ensure sustained customer engagement and market penetration.

### Measures and capabilities for implementing and sustaining key success factors

Our qualitative study not only identified category-specific success factors for patient-facing DHT companies but also explored the critical measures and capabilities required to achieve and sustain them. Table 3 provides an overview of the key measures necessary for attaining internal success factors. To further validate our findings, we conducted a follow-up survey among the same group of executives and founders we initially reached out to for interviews. This included both those who participated in the interviews and those who had been contacted but did not take part. In total, 27 executives and founders responded to the survey, including 23 interviewees. Survey participants selected the identified measures they considered most important, offering a quantified perspective on their perceived relevance. The “Mentions” column in Table 3 indicates the number of survey participants who selected each measure after identifying the corresponding success factor in their responses. This structured assessment provides additional insight into which measures executives and founders consider most critical for scaling patient-facing DHT companies. Participant demographics for the follow-up survey can be found in Supplementary Material 5.

**Table 3:** Measures and capabilities for implementing and sustaining key internal success factors. Mentions represent the number of survey participants who selected each measure after identifying the corresponding success factor in their responses

Segment	Factor	Measures and capabilities	Mentions
**1. Business Models**	a. Business model flexibility	⇒ Rapid experimentation and market adaptation	10 (37%)
		⇒ Tailoring to diverse customer needs	5 (19%)
		⇒ Pivoting quickly from failing models	5 (19%)
	b. Internationali-zation strategy	⇒ Leveraging partnerships for market entry	6 (22%)
		⇒ Adapting to local market conditions	4 (15%)
		⇒ Strategic market selection and timing	3 (11%)
	c. Strategic market positioning	⇒ Clear communication of intended use	7 (26%)
		⇒ Start in non-medical or less regulated markets	2 (7%)
	d. Sales and marketing strategy	⇒ Leveraging partnerships for scaled sales	9 (33%)
		⇒ Direct-to-consumer awareness and trust building	5 (19%)
		⇒ Data-driven customer management and optimization	4 (15%)
**2. Product & Service**	a. Product-market fit	⇒ Clear unmet need identification	14 (52%)
		⇒ Extensive market research and validation	11 (41%)
	b. Regulatory certification	⇒ Building clinical evidence and meeting quality standards	7 (26%)
		⇒ Strategic regulatory planning	3 (11%)
		⇒ Ensuring compliance with data privacy regulations	2 (7%)
	c. Quality and performance	⇒ Providing unique solutions tailored to customer needs	6 (22%)
		⇒ Building trust through authenticity and research	5 (19%)
		⇒ Self-regulation for continuous improvement	3 (11%)
**3. Employees**	a. Leadership experience	⇒ Industry-specific leadership experience	3 (11%)
		⇒ Determination and ability to execute	3 (11%)
	b. Diversity of expertise within employees	⇒ Ensuring a wide range of capabilities	1 (4%)
		⇒ Assembling the team based on problem-solving needs	1 (4%)
	c. Employee alignment with the company’s vision	⇒ Careful selection of passionate team members	2 (7%)
		⇒ Transition from hero culture to team culture	1 (4%)

#### Internal success factors measures and capabilities

1. Business model
  - *Business model flexibility*
    The flexibility of a company’s business model is essential for adapting to rapid shifts in market demands. Agile methodologies that support quick experimentation and adaptation are crucial for success. As noted by one of our interviewees, rapid testing to find a sustainable model can involve looking beyond traditional healthcare settings:
    *“It’s important to experiment quickly to find a sustainable commercial model. Sometimes*,* this may involve developing solutions outside the healthcare system. While that might seem unconventional*,* relying heavily on hospital settings can be limiting. Creating a solution that works in other contexts—such as direct-to-consumer—offers the flexibility needed to identify the most effective business model.” [I7]*
    This iterative approach allows companies to make product and strategy adjustments closely aligned with customer feedback. Interviewed DHT companies emphasized the importance of tailoring offerings to meet diverse customer needs:
    *“We’re not very rigid when it comes to the business model because […] different clients*,* especially when it comes to different sizes of clients and different markets*,* have different expectations. We always try to make it work*,* so we don’t lose the deal unless it is […] a loss-making deal for us. If there is some potential for benefit and revenue*,* we try to make it work*,* even if we [make] exceptions when it comes to the pricing and the business model.” [I26]*
    The ability to pivot swiftly away from ineffective models minimizes resource misallocation and enables companies to focus on strategies with higher growth potential:
    *“[Our business model] evolved maybe ten times. We started with a B2C model*,* then we wanted to sell to individual physicians*,* then we wanted to sell to clinical trial CRO companies*,* then we wanted to sell our services to […] electronic health record [companies]*,* and then we wanted to sell to health systems. […] A few years in*,* we received an email from [a large health insurance group]*,* and they said*,* look*,* we’re looking for that kind of technology that we could white label and integrate with our application for members. And that’s what we did.” [I12]*
  - *Internationalization strategy*
    Internationalization is a key driver of growth, necessitating a well-crafted strategy for new market entry. Companies often evaluate market size, competitive intensity, and penetration to optimize market choice and timing:
    *“The US is about 45–50% of the global healthcare IT spend. Therefore*,* it makes sense to go there […]. When we started*,* the business recognized that it was a much more tractable market to tackle than the UK or Europe. So*,* we really just focused on that first.” [I10]*
    In healthcare, companies benefit from localizing their approach to account for varying cultural, economic, and regulatory conditions:
    *“We started in Germany*,* we expanded to the UK*,* to demonstrate we can operate in different regulatory environments*,* cultural settings*, etc.,* the product works there. Which is a sort of a small step towards the US.” [I17]*
    Strategic partnerships with established organizations can also allow market entry, providing access to networks and reducing initial costs:
    *“For now*,* we’re in France*,* where we are obviously well developed. We started […] in Belgium*,* the Netherlands and Spain*,* and the US*,* through our pharma partnership at the time.” [I18]*
  - *Strategic market positioning*
    Effective market positioning, especially in the DHT space, often involves beginning in less-regulated consumer markets, generating initial revenue, and building regulatory expertise before expanding into highly regulated healthcare sectors:
    *“It was crucial not to position ourselves as a medical solution; otherwise*,* we would not have had the chance to succeed. […] The last thing the [physician community] wants is for someone to come along*,* introduce efficiencies*,* and disrupt the status quo. Therefore*,* we deliberately chose not to enter the medical field at the outset.” [I1]*
    This phased approach also avoids prematurely investing in certifications that may not align with market demands. As another participant noted: *“A few years ago*,* we decided to certify a product as a medical device. This ended up being a big mistake because our clients did not want to adopt a service that is a medical device […] we had to revert this.” [I26]*
    Communicating a product’s intended use is another aspect of effective market positioning, as it helps avoid regulatory barriers and fosters user trust:
    *“We can’t claim*,* oh*,* well*,* we can detect Covid […]. We tell you that your temperature is elevated*,* your respiration is elevated*,* and your [heart rate variability] (HRV) has plummeted. […] being clear about what it is that we do and being in spaces and domains and markets that are not regulated.” [I28]*
  - *Sales and market strategy*
    Several DHT companies prioritize direct-to-consumer marketing through digital ads and TV to effectively reach their target segments:
    *“We have predominantly done marketing in the digital channels like Meta and Google and then in TV. And actually*,* TV has worked quite well for us because*,* for the hypertension target group*,* [our customer’s] average age is around 55.” [I20]*
    Strategic partnerships also scale sales operations, enabling a more extensive market reach through collaboration with larger organizations:
    *“We leveraged on our [top pharma] partners’ salesforce […] so that we could have doctors willing to integrate our product inside their workflow.” [I18]*
    Data-driven customer management further refines engagement strategies and improves retention, helping companies optimize communication and relationships with their customers:
    *“Once you have enough [customers] in the customer base […]*,* you look into churn rate*,* […] average lifetime value*,* […] conversion rate to win*,* conversion rate to lost […]*,* and then you can compute a variety of different KPI’s that will tell you if it makes sense to sell to this [customer] group.” [I12]*

2. Product& service
  - *Product market fit*
    In the product and services domain, achieving product-market fit is essential for scaling and is even seen by some as “all that matters,” as stated by an interviewee. Successful DHT companies demonstrate an understanding of unmet market needs —the most frequently mentioned measure in the survey—allowing them to develop solutions that address specific, real-world challenges. For instance, one interviewee shared:
    *“I was a volunteer in a care organization where people lived with a severe intellectual disability. [A patient] couldn’t express his emotions because of his intellectual disability*,* but he was suffering from pain. And I thought*,* why can we not measure his emotions with the sensor? So*,* it starts with a clear unmet need with a problem that you try to tackle.” [I13]*
    Early, comprehensive market research is crucial in identifying these unmet needs, refining product features, and minimizing the risk of misalignment with market demands. Some CEOs regretted not investing enough in early-stage market research, which delayed their path to product-market fit, while others highlighted how foundational it was to their current success:
    *“I worked as an astronomer for a while*,* developed some technology*,* and thought I could apply that to medical imaging. And you […] have all sorts of preconceptions about how the world is*,* and you decide that you can put your technology in a certain way. And it turns out that your preconceptions*,* or at least my preconceptions of the world*,* were quite wrong. But you only find that by really testing what you’re doing and talking to a lot of people.” [I10]*
  - *Regulatory certification*
    Regulatory certification is a critical requirement, particularly for companies in sectors like Patient Monitoring, Digital Diagnostics, and Digital Therapeutics. Proactive regulatory planning enables these companies to streamline compliance processes, strategically timing their entry into various regulatory classifications to minimize delays and reduce compliance costs.
    *“You also need a regulatory strategy and understanding what features or capabilities you introduce will move you up regulatory bandings […]. To date*,* we’re still very careful not to move beyond class one. And we know there are some features we will want to introduce in the future that would move us up to a class two or class three.” [I5]*
    To obtain certification, companies must meet clinical evidence standards and quality benchmarks, which also helps build trust among healthcare providers and end-users and enables growth.
    *“You cannot go to the market and sell [your solution]. First of all*,* you have to go through this certification part and collect all the clinical evidence.” [I25]*
    Given the sensitivity of health data, compliance with data privacy regulations, such as GDPR, is essential; breaches in these areas can lead to both reputational and legal consequences.
    *“And then also*,* from the data privacy side*,* that we fully comply with the GDPR because I think that was always what customers wanted to hear. And then you need to*,* of course*,* have the supporting documentation and evidence.” [I22]*
  - *Quality and performance*
    Maintaining high quality and performance is key to long-term success, as trust among users is essential. DHT companies can foster this trust by demonstrating authenticity and transparency throughout their research and development processes.
    *“It’s not really brand recognition*,* name recognition*,* but what’s the word? It is authenticity. And a large*,* readily available body of research […] on [your company’s technology]. So that has meant that we have always had more […] attention than you would normally get for a company our size.” [I11]*
    Tailoring DHTs to meet specific customer needs enhances perceived value and differentiates a company from its competitors. To gain widespread adoption, products must be precise, user-friendly, compact, and affordable. As one CEO noted:
    *“A winning [solution] needs to be accurate; it needs to be small; it needs to be easy to use*,* and then it needs to be affordable. And if we fail even in one of those*,* then we can pack our bags and go home.” [I21]*
    Continuous improvement through self-regulation helps companies ensure their products remain aligned with market needs and maintain their quality over time.
    *“[We are] self-regulating based on self-interest. It is in our self-interest to build the best product possible.” [I29]*
3. Employees
  - *Leadership experience*
    Leadership experience, particularly in healthcare and digital technology, is crucial in navigating the industry’s complex regulatory and market challenges. Leaders with domain-specific backgrounds bring valuable insights and strategic capabilities to their organizations, as noted by one of our interviewees:
    *“[The company] was founded by four people*,* including myself*,* all of whom came from digital health. We had been working in this space for ten years*,* so I think experience is one success factor.” [I3]*
    A cohesive leadership team built on trust and shared goals strengthens strategic alignment and operational effectiveness. As one respondent shared:
    *“We have a team that has worked together before our leadership. There’s seven of us for whom this is a third company that has been really key.” [I29]*
    Effective leadership also involves resilience and a determined approach to overcoming challenges, which is essential in the often-unpredictable digital health landscape:
    *“The resilience of the founding team and determination to push through any challenge that comes up is very important because when we started in 2019*,* there was no clear path to reimbursement.” [I19]*
  - *Diversity of expertise among employees*
    A diverse skill set within the employee base enhances a company’s adaptability and problem-solving capacity. Cross-functional teams with expertise in technology, healthcare, marketing, and regulation can address the multifaceted challenges in digital health more effectively. Employing a range of capabilities allows teams to approach problems holistically and fosters innovation:
    *“Getting the right team members on board with the right capabilities within a wide range of topics*,* including innovation*,* clinical competence*,* tech development*,* commercial competence.” [I14]*
    Organizing teams around specific problem-solving needs further cultivates a results-oriented culture where each member contributes unique value to organizational goals:
    *“We didn’t start off as hammers looking for nails. We didn’t say we are radar engineers. Let’s go solve healthcare. We started with the problem first; then*,* we assembled the team to go solve that problem. And that’s been incredible.” [I29]*
  - *Employee alignment with the company’s vision*
    Aligning employees with the company’s vision is essential to fostering sustained growth. Carefully selecting team members who are passionate about healthcare and technology enhances motivation and commitment within the company. One CEO emphasized the importance of reinforcing the company mission:
    *“You articulate the vision*,* and then you repeat it. And it’s self-selecting because people join the company because they believe in the mission. And so*,* people get reinforced about the mission that they’re on.” [I28]*
    As companies scale, transitioning from a “hero culture,” where individual contributions are disproportionately highlighted, to a team-oriented culture is essential for sustainable growth. This shift ensures that the organization’s success is driven by a well-integrated team rather than a few individuals:
    *“Internally*,* it’s culture. You have to shift from a hero culture to a team-based culture. Initially*,* individuals use tenacity and grit to drive success*,* but as you grow*,* you need teams that are aligned. It’s not one person ruling the day anymore; you need the team. You move from tribal knowledge to more operating rhythms.” [I6]*

#### External success factors measures and capabilities

Achieving external success factors is essential for patient-facing DHTs, as these companies must engage various stakeholders, including patients, healthcare providers, payers, pharmaceutical companies, and hospitals. Effective engagement requires strategic measures and capabilities, as summarized in Table 4, which outlines the key segments and corresponding success factors.

1. Healthcare system
  - *Health impact and proof validation*
    Proof of health impact and clinical validation are foundational in the healthcare system. DHT companies must establish clinical evidence through rigorous studies that demonstrate both short- and long-term impacts on patient outcomes.
    *“The bit that we don’t modify is the clinical care pathways or programs because the thing that we are most focused on over the next twelve months is our evidence-generation strategy. So*,* really proving our clinical outcomes and our health economics. Therefore*,* we need as many people as possible to move through a standardized care pathway so we can gather data.” [I7]*
    Clinical trials serve as a valuable method for establishing trust among healthcare providers and regulatory bodies. By undertaking clinical trials, DHT companies can showcase their commitment to transparency and demonstrate the credibility of their technologies:
    *“The way we are using that is to say*,* look*,* you can trust us. We are one of the really honest companies about this and most advanced companies. And you can see that because we’re going through clinical trials with our technology*,* we’re actually opening us up entirely.” [I11]*
  - *Regulatory environment and policy framework*
    Navigating the regulatory environment requires strategic anticipation. For many DHT companies, engaging with reimbursement models, such as DiGA in Germany or PECAN in France, is essential for securing market access and facilitating adoption within healthcare systems:
    *“There has been a payment model in Sweden that has allowed digital care providers to provide health care without requiring specific procured contracts. So*,* you can essentially apply to be part of this model. So over 90% of our patients today are publicly financed*,* and they pay co-payments for consultations.” [I20]*
    Balancing regulatory compliance with scalability is also critical. Companies must meet healthcare regulations, such as data privacy and certification requirements while maintaining growth potential:
    *“Many of our suppliers are looking to do the minimum to achieve a certification. Many of our channels are looking to do far more because they’re very concerned about reputational brand damage if they do something wrong.” [I10]*
    Remaining proactive about regulatory changes helps companies stay competitive by enabling quick adjustments to compliance requirements, which, as one interviewee noted, often evolve:
    *“B-pharm*,* the regulatory authority in Germany*,* […] have very strict requirements on your security*,* infrastructure*,* data protection and so on. And the requirements constantly develop and change.” [I19]*
  - *Financial impact proof and validation*
    Financial impact validation is crucial in convincing stakeholders of the economic benefits of DHTs. Demonstrating cost savings through data-backed case studies reinforces the financial value of these technologies—a point emphasized by one interviewee:
    *“We showed lowering down costs. We have a very beautiful study in the Netherlands with children with asthma where we have shown that we cut off cost management cost for this patient by 80%.” [I4]*
    By translating clinical benefits into measurable financial outcomes, DHT companies align their value propositions with healthcare systems’ objectives, highlighting the solutions’ return on investment:
    *“Lots of people will tell you about the clinical benefits of an AI product. And although that’s really what drives me and many of the people that work for me*,* it isn’t what drives a purchasing decision by a healthcare organization. […] For all of these fantastic clinical benefits*,* you have to be able to express them in financial terms.” [I10]*
2. Customers
  - *Customer feedback*
    Active customer engagement is crucial for gathering feedback, validating product ideas early in the development phase, and refining features to meet user needs:
    *“I think another one is spending a lot of time really listening. […] We take a lot of time to make sure our product team really talks as much as they can to our end users.” [I5]*
    This continuous dialogue helps DHT companies enhance usability and address pain points while prioritizing customer support strengthens trust and encourages positive experiences:
    *“When it comes to customers*,* there are so many things that can potentially not work in a private cloud like ours […] we decided from the beginning that customer support is a very high priority.” [I9]*
  - *Customer awareness raising*
    Raising awareness involves credibility-building and educational campaigns. Key opinion leaders (KOLs) and ambassadors play a significant role in establishing trust, especially in markets that may be resistant to new technologies:
    *“Having ambassadors in the field is crucial. Champions who promote our product can make a big difference. It’s important to have strong sales and business development alongside technology.” [I22]*
    Educational campaigns, including webinars, whitepapers, and social media, broaden understanding of a product’s value, helping to overcome adoption barriers:
    *“We did a lot to kind of educate the market about HRV. You know*,* we talk about it on podcasts*,* and we would put it on our website*,* and we would write white papers about it.” [I28]*
    Proactively addressing barriers to adoption, such as clarifying benefits and countering misconceptions, reduces friction for new customers, particularly in healthcare, where resistance to technological change is often high:
    *“The challenge is the pace of the market. So long-term care in the Netherlands is slow*,* it’s very slow. And so*,* they don’t easily adopt change […] Technology is new. They also see it as cold*,* so they provide warm care*,* and technology is cold care. There’s a paradigm shift needed to also prepare for the future and to provide the care that’s needed for the future.” [I13]*
  - *Regional market size and customer behavior*
    Understanding regional market dynamics enables companies to prioritize expansion in areas with high demand and positive attitudes toward digital health innovation:
    *“One of the keys is that it’s an area that scales well because it’s a huge market with high demand—weight loss and obesity treatment. It’s a massive global problem.” [I3]*
    Acknowledging regional variations in customer behavior helps DHT companies tailor their strategies to maximize engagement and adoption:
    *“It turned out that Asia experienced the strongest growth initially*,* and our first clients came from there. Europe followed*,* but it was much more conservative*,* particularly in the German-speaking and French-speaking regions.” [I1]*
3. Partnerships
  - *Collaboration with larger organizations*
    Collaborating with larger organizations provides strategic advantages, allowing DHT companies to leverage intellectual property (IP) to align with partners’ goals. Such partnerships facilitate co-development, technology transfer, and broader distribution:
    *“Partnership working was key with the large [Original Equipment Manufacturers] (OEMs)*,* the kind of the Philips’ of the world*,* trying to make sure that we’re solving a problem that they can’t solve and being patient whilst it takes them a long time to build a relationship.” [I10]*
    Paid partnerships establish stability and long-term commitment, as partners have a vested interest in the success of the DHT company:
    *“In that phase of the company*,* I involved a lot of care organizations in the Netherlands to co-finance the development. So instead of doing free pilots*,* I said*,* it’s an important problem; you have identified it as well*,* and I would like you to be involved in the development*,* but you have to pay for it.” [I13]*
  - *Investor backing and fit*
    Investor backing is critical for growth for some DHT companies. Choosing investors who provide “Smart Money,” financial support along with industry insights and strategic guidance, can significantly influence a company’s success trajectory:
    *“We had very good investors that […] could help us initially in the product development*,* software-related questions*,* and then also later on; they could get us connections to the healthcare industry.” [I22]*
    Alignment between investors and founders is essential for ensuring long-term success, as shared values and vision foster stability and strategic coherence:
    *“Choosing the right investors*,* I would say*,* is also important in terms of them being aligned with one’s personal values.” [I14]*
    Leveraging networks can help to find funding and partnerships to further support sustainable growth:
    *“We’ve been relatively successful with fundraising. And the success factors there are still*,* I would say*,* sadly*,* network*,* and we’re female-founded*,* so it’s myself and another woman*,* and we had to leverage the network of*,* you know*,* anyone that we could.” [I7]*

**Table 4:** Measures and capabilities for implementing and sustaining key external success factors. Mentions represent the number of survey participants who selected each measure after identifying the corresponding success factor in their responses

Segment	Factor	Measures and capabilities	Mentions
**1. Healthcare System**	a. Health impact proof and validation	⇒ Building clinical evidence and health economics	8 (30%)
		⇒ Leveraging clinical trials for trust and validation	1 (4%)
	b. Regulatory environment and policy framework	⇒ Staying informed and proactive on regulatory changes	5 (19%)
		⇒ Leveraging reimbursement models for market access	4 (15%)
		⇒ Balancing regulatory compliance with scalability	2 (7%)
	c. Financial impact proof and validation	⇒ Demonstrate cost savings with data-backed case studies	8 (30%)
		⇒ Translate clinical benefits into financial impact	7 (26%)
**2. Customers**	a. Customer feedback	⇒ Regular engagement with customers	3 (11%)
		⇒ Validating product ideas early through customer input	3 (11%)
		⇒ Prioritizing customer support	2 (7%)
	b. Customers awareness raising	⇒ Leverage key opinion leaders and ambassadors for credibility	5 (19%)
		⇒ Educate the market through multi-channel campaigns	5 (19%)
		⇒ Address resistance to new technologies	1 (4%)
	c. Regional market size and customer behavior	⇒ Prioritize markets with high demand and receptive attitudes towards innovation	5 (19%)
		⇒ Adapt go-to-market strategies to regional customer behavior	5 (19%)
**3. Partner-ships**	a. Collaboration with larger organizations	⇒ Establish paid partnerships for long-term commitment	4 (15%)
		⇒ Leverage intellectual property to align with larger partners	1 (4%)
	b. Investor backing and fit	⇒ Ensuring investor and founders vision align	10 (37%)
		⇒ Choosing investors who offer “Smart Money”	6 (22%)
		⇒ Leveraging network for funding	4 (15%)

## Discussion

**Fig. 3:**
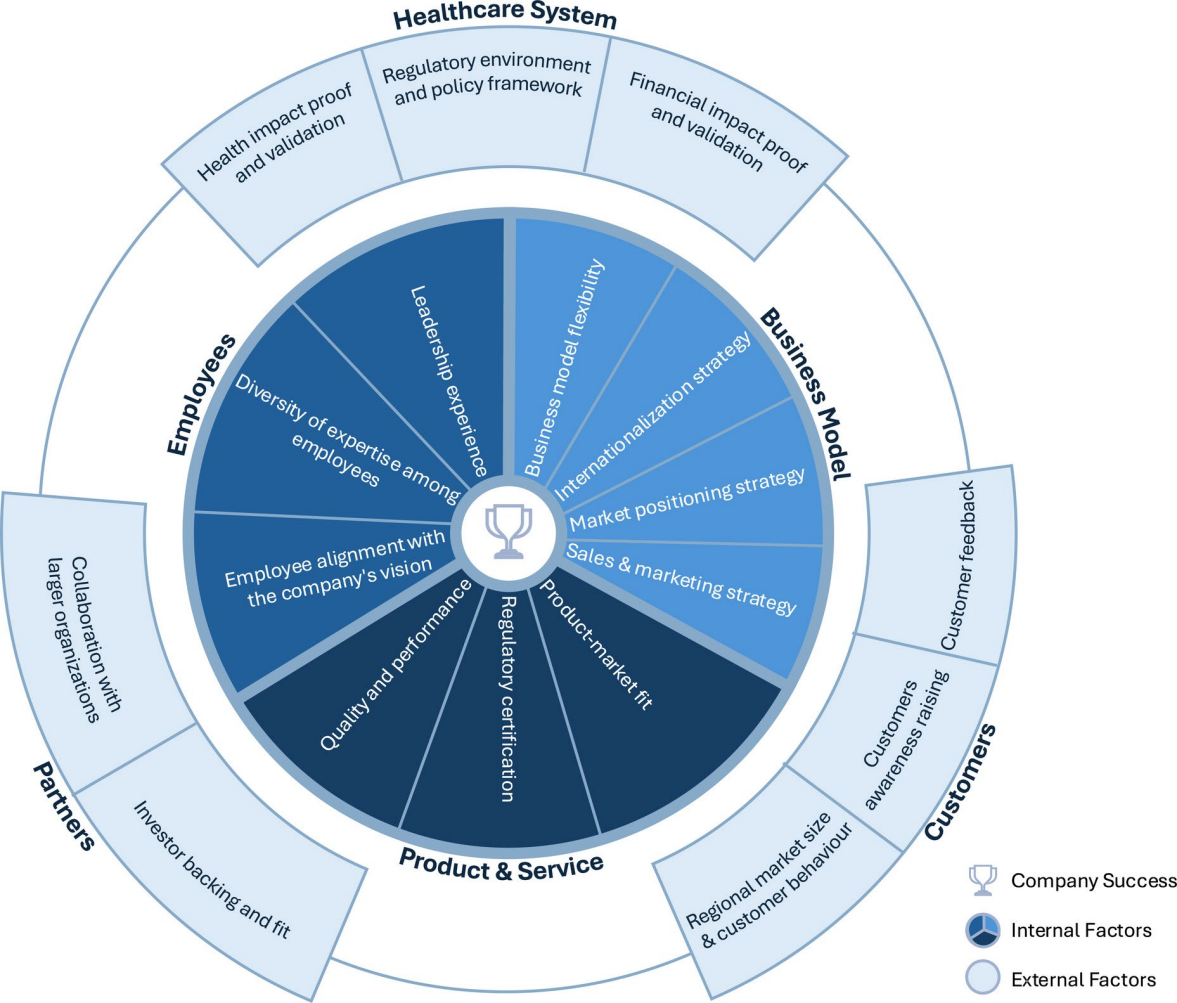
Patient-facing digital health company success thematic map: this model presents an overview of essential success factors for digital health companies. The inner circle illustrates internal success factors within a DHT company, while the outer circle highlights external success factors shaping the broader business environment

This study reveals the complex landscape of success factors essential for scaling patient-facing DHT companies, highlighting internal factors (e.g., business model flexibility and regulatory certification) and external factors (e.g., customer awareness and partnerships). As shown in Fig. 3, our thematic map of these factors reinforces the need for category-specific strategies rather than a one-size-fits-all approach. Each DHT category requires strategies tailored to its operational, regulatory, and end-user demands, aligning with broader challenges, such as supporting healthcare resilience amid aging populations and workforce shortages across Europe.

For example, in the Health & Wellness DHT category, adaptability is important due to its consumer-driven nature. One executive highlighted how their company developed a flexible business model, strategically positioning its solution in the lifestyle segment to avoid resistance from heavily stakeholder-driven sectors. This allowed them to build a user base by focusing on holistic wellness rather than direct medical claims, aligning their service with consumer needs for a non-clinical solution. By positioning themselves outside of traditional healthcare, Health & Wellness companies can reach consumers directly, helping to relieve healthcare burdens by promoting proactive, preventive health measures.

For Patient Monitoring and Digital Therapeutics companies, regulatory strategy and product-market fit were critical factors. Some executives described the challenges of establishing clinical utility in regulated environments. To navigate these barriers, companies in these categories often adopt rigorous evidence-generation strategies, underscoring the need for tailored approaches that recognize the high stakes of regulatory compliance for medical-grade solutions.

A recurring success factor across all DHT categories is demonstrating tangible healthcare impact and validation. To achieve meaningful growth, DHTs must move beyond being perceived as “nice to have” and establish themselves as indispensable healthcare system components. This necessitates robust clinical evidence generated through rigorous studies that substantiate both short- and long-term improvements in patient outcomes. Early-stage DHT companies can use publicly available data to formulate preliminary assumptions, signaling their commitment to addressing critical healthcare needs. Over time, these assumptions can be rigorously tested and validated through systematic trials and real-world evidence, allowing companies to build a compelling case for their role in enhancing healthcare resilience and efficiency.

Our findings suggest that digital health requires more than a generalized scaling strategy. Instead, aligning strategic priorities with each category’s unique operational, regulatory, and end-user requirements can position DHT companies to effectively address healthcare challenges across Europe.

### Theoretical contribution

Our findings offer a deeper understanding of success factors within each DHT category, critically examining the limitations of generalized growth strategies for digital health companies. While our previous systematic literature reviews identify broad success factors for growth-stage digital companies, such as market demand, user-centered design, product innovation, financial viability, technology integration, and customer feedback [33], our study refines these factors specifically for patient-facing DHTs. Through our interview study, we provide more precise insights into the strategic imperatives needed to address the unique challenges faced by companies operating within different DHT categories. This nuanced analysis bridges the gap between theoretical success factors and actionable strategies, offering a clearer, category-specific perspective on scaling patient-facing DHTs.

While prior research has explored clinical pipelines, validation processes, and system integration of DHTs [52–54], fewer studies have addressed the specific factors and measures required for scaling DHTs within Europe’s diverse regulatory and market environments. Our study contributes to the broader conversation on scaling digital health technologies by offering a more detailed understanding of the category-specific challenges DHT companies face in Europe’s regulatory and market contexts. This work expands the theoretical discourse on DHT adoption and growth, providing more targeted insights into the success factors that enable digital health companies to thrive across diverse healthcare environments [55–59].

In doing so, our results partially echo classic management theories while also bringing some unique considerations of DHT delivery. For instance, Barney’s Resource-Based View positions distinctive competencies (e.g., regulatory certification) as a key source of competitive advantage [60]; our study confirms this but also shows how DHT providers must continuously demonstrate clinical impact to maintain relevance in a fast-evolving regulatory space. Similarly, Porter’s competitive strategies remain relevant for positioning and differentiation, yet we find that patient-facing DHTs cannot simply rely on cost or product features alone; they must also secure trust through validated outcomes [61]. Finally, while Kaplan and Norton’s Balanced Scorecard calls for a multi-perspective approach to performance, the need for clinical, financial, and regulatory milestones is particularly heightened in healthcare, indicating that broader management frameworks may require added layers of evidence-based metrics to capture success in this domain [62].

By situating our findings within both digital health and broader management perspectives, this study enriches the theoretical discourse on DHT adoption and growth, offering a more nuanced roadmap for companies and stakeholders seeking to navigate Europe’s complex healthcare ecosystems.

### Managerial contribution

Our findings offer actionable insights for founders, executives, and investors, promoting a comprehensive, balanced approach to growing patient-facing DHTs that aligns with the distinct needs of each category. For founders and executive teams, the study emphasizes the importance of adapting strategies to each DHT type’s operational demands. For instance, leaders in Health & Wellness may benefit from a flexible business model that accommodates varied consumer needs and responds to rapidly shifting market dynamics. In contrast, leaders in Digital Therapeutics may need to prioritize building dedicated teams focused on resource allocation for regulatory compliance and validating clinical impact. This nuanced understanding enables DHT companies to tailor their approaches based on clear, category-specific requirements, fostering sustainable growth and resilience to sector-specific challenges. By detailing the capabilities and measures taken by successful companies in this field, our study provides concrete guidelines for achieving key success factors.

For investors and stakeholders, our findings identify key areas for strategic support and collaboration with patient-facing DHT companies. By understanding the specific success factors and key capabilities required for each DHT category, investors can make more informed decisions about resource allocation and support, particularly in areas like regulatory compliance or customer engagement. The measures and capabilities detailed in this study provide practical strategies for establishing and maintaining a strong growth trajectory, offering investors more precise recommendations on scaling DHT companies. Our findings are also valuable for other stakeholders, such as health insurers and healthcare providers. It enables them to recognize what is essential for DHT companies to succeed before entering collaborations, thus fostering mutual benefit and long-term success.

### Limitations and future research recommendations

This study has several limitations that should be considered. Firstly, our focus on European companies may limit the generalizability of the findings to other regions with different healthcare regulatory landscapes and market dynamics. We focused on Europe because of the diverse regulatory landscape, languages, and business dynamics. However, future studies could expand this research to include perspectives from DHT companies in North America, Asia, and other regions to allow for cross-regional comparisons.

Another limitation of our study is that it does not account for how the importance of success factors may shift as a company progresses through different stages of growth. While we identified critical success factors across DHT categories, their relative importance evolves as companies mature, with early-stage companies potentially prioritizing factors like product-market fit and more established companies focusing on regulatory compliance and scaling partnerships. Future research could explore this dynamic, examining how success factors vary across growth stages to provide a more nuanced understanding of the strategies needed for companies at different development phases.

While all the identified success factors were emphasized by several executives from advanced digital health companies, they are not universally applicable and should be evaluated based on a company’s unique context. For instance, collaboration with larger organizations can be a double-edged sword. While such partnerships may accelerate short-term growth, they often impose constraints that limit a company’s ability to form additional partnerships with other players in the market.

Among the executives and founders we interviewed, 28% (*n* = 8) were based in the United Kingdom. While our study achieved broad geographic coverage across Europe, this concentration may have introduced a bias toward the United Kingdom as the most represented country. Future research could strive for a more balanced geographic distribution, incorporating underrepresented countries such as Spain and Portugal to provide a more comprehensive perspective.

While Fig. 3 provides an overview of the discussed success factors, further research could refine and expand this model into a more generalizable framework. One potential approach would be to conduct a Delphi study, engaging a panel of digital health experts, industry leaders, and policymakers to iteratively evaluate and validate the critical success factors identified in this study. Through a structured consensus-building process, this method could help establish a robust, evidence-based framework applicable across different digital health contexts.

Finally, the digital health field is rapidly evolving, and the success factors identified in this study may change over time as new technologies and market conditions emerge. Longitudinal studies will be valuable in capturing how the relevance of these factors shifts over the coming years, allowing stakeholders to adjust their strategies in response to future industry developments.

## Conclusion

This study identifies key success factors for scaling patient-facing DHTs, emphasizing tailored strategies for each category. Critical drivers such as business model flexibility, regulatory compliance, and validation of health and financial impact, combined with external factors like customer awareness and strategic partnerships, are essential for sustainable growth. By equipping DHTs to enhance healthcare services and address Europe’s growing healthcare challenges, this research provides a practical basis for founders, executives, and healthcare stakeholders to accelerate adoption and improve system resilience.

## Supplementary Information

Supplementary Material 1.

Supplementary Material 2.

Supplementary Material 3.

Supplementary Material 4.

Supplementary Material 5.

Supplementary Material 6.

## Data Availability

The data supporting this study’s findings are available from the corresponding author upon request.

## Abbreviations

NCDs: Non-Communicable Diseases
DHTs: Digital Health Technologies
DTx: Digital Therapeutics
COREQ: Consolidated Criteria for Reporting Qualitative Research
FTEs: Full-Time Employees
DiGA: Digital Health Applications (Germany’s program for prescribing and reimbursing digital health tools)
KOLs: Key Opinion Leaders
GDPR: General Data Protection Regulation
WHO: World Health Organization
B2C: Business-to-Consumer
CRO: Contract Research Organization
EHR: Electronic Health Record
PECAN: A reimbursement model in France
OEMs: Original Equipment Manufacturers
HRV: Heart Rate Variability
