# Supplementary material for: Success factors and measures for scaling patient-facing digital health technologies from leaders’ insights
Source: BMC Health Serv Res. 2025 May 1;25:632. doi: 10.1186/s12913-025-12748-z (PMC12046742; doi:10.1186/s12913-025-12748-z)
Supplement: Supplementary file 1 — Supplementary Material 1. [file 12913_2025_12748_MOESM1_ESM.docx]

**Multimedia Appendix 1: COREQ checklist**

Table S1: Consolidated Criteria for Reporting Qualitative Research (COREQ) checklist for semi-structured interviews and detailed reporting summary

| **No.** | **Item** | **Guide questions/ description** | **Reporting** |
| --- | --- | --- | --- |
| **Domain 1: Research team and reﬂexivity** | | | |
| *Personal Characteristics* | | | |
| 1. | Interviewer/facilitator | Which author/s conducted the interview or focus group? | - Estelle Pfitzer |
| 2. | Credentials | What were the researcher’s credentials?  *E.g., PhD, MD* | - Estelle Pfitzer: M.Sc., B.Sc. - Odile Florence Giger: M.Sc., B.Sc. - Christoph Kausch: PhD, M.Sc. B.Sc. - Tobias Kowatsch: Prof., PhD, M.Sc., M.Sc., Dipl.-Inf. (FH) |
| 3. | Occupation | What was their occupation at the time of the study? | - Estelle Pfitzer: Research analyst - Odile Florence Giger: Research assistant - Christoph Kauch: Managing partner - Tobias Kowatsch: Professor |
| 4. | Gender | Was the researcher male or female? | - Estelle Pfitzer: Female - Odile Florence Giger: Female - Christoph Kauch: Male - Tobias Kowatsch: Male |
| 5. | Experience and training | What experience or training did the researcher have? | - Every member of the research team is experienced and has received training in conducting qualitative research |
| *Relationship with participants* | | | |
| 6. | Relationship established | Was a relationship established prior to study commencement? | - The research team conducting the interviews had no previous connections with the participants and had not interacted with them prior to the interviews. |
| 7. | Participant knowledge of the interviewer | What did the participants know about the researcher? *E.g., personal goals, reasons for doing the research* | - The objective of this study was shared via the outreach email. Additionally, the study's objectives and research intent were emphasized at the beginning of each interview |
| 8. | Interviewer characteristics | What characteristics were reported about the interviewer/ facilitator? *E.g., bias, assumptions, reasons, and interests in the research topic* | - At the start of each interview, during the introduction phase, the participant was informed about the interviewer’s role and current occupation. Additionally, information about the interviewer’s characteristics and professional background was made available on the institute's website |
| **Domain 2: Study design** | | | |
| *Theoretical framework* | | | |
| 9. | Methodological orientation and Theory | What methodological orientation was stated to underpin the study? *E.g., grounded theory, discourse analysis, ethnography, phenomenology, content analysis* | - The study was based on a qualitative research methodology using in-depth semi-structured interviews, employing inductive thematic analysis combined with deductive analysis to develop and refine a preliminary codebook, as detailed in the "Methods" section |
| *Participant selection* | | | |
| 10. | Sampling | How were participants selected? *E.g., purposive, convenience, consecutive, snowball* | - Participants in this study were selected purposively. Using PitchBook, we focused on revenue-generating companies with over 20 employees targeting NCDs. This approach ensured the inclusion of executives and founders from established and diverse European patient-facing DHT companies |
| 11. | Method of approach | How were participants approached? *E.g., face-to-face, telephone, mail, email* | - Participants were approached via email, utilizing contact information obtained through PitchBook and the companies’ official websites. Initial outreach emails provided an overview of the study, its objectives, and an invitation to participate in a virtual interview. Follow-up emails were sent to clarify any questions and schedule the interviews |
| 12. | Sample size | How many participants were in the study? | - 29 participants took part in the study |
| 13. | Non-participation | How many people refused to participate or dropped out? Reasons? | - Four participants withdrew from the study. The reasons for discontinuation included time constraints and being unresponsive despite initially agreeing to participate |
| *Setting* | | | |
| 14. | Setting of data collection | Where was the data collected? *E.g., home, clinic, workplace* | - All interviews were conducted via video calls |
| 15. | Presence of non-participants | Was anyone else present besides the participants and researchers? | - No one other than the participant and the researcher was present |
| 16. | Description of sample | What are the important characteristics of the sample? *E.g., demographic data, date* | - All participants held C-level positions at digital health companies and met the eligibility criteria for study inclusion. Detailed information on the participants’ socio-demographic and professional characteristics can be found in Table 1 |
| *Data collection* | | | |
| 17. | Interview guide | Were questions, prompts, guides provided by the authors? Was it pilot tested? | - The interview framework underwent pilot testing before the first interview. Participants were informed that no advance preparation was necessary. The structure and agenda of the interview were presented at the outset |
| 18. | Repeat interviews | Were repeat interviews carried out? If yes, how many? | - No interview was repeated |
| 19. | Audio/visual recording | Did the research use audio or visual recording to collect the data? | - All interviews were audio-recorded with the participants’ consent |
| 20. | Field notes | Were ﬁeld notes made during and/or after the interview or focus group? | - Field notes were not taken |
| 21. | Duration | What was the duration of the interviews or focus group? | - Interviews lasted, on average, 28 minutes and ranged between 21 and 40 minutes |
| 22. | Data saturation | Was data saturation discussed? | - The researchers deliberated on data saturation |
| 23. | Transcripts returned | Were transcripts returned to participants for comment and/or correction? | - Transcripts were not returned to participants for comment or correction. To ensure accuracy, the researchers thoroughly reviewed all transcripts against the original recordings |
| **Domain 3: analysis and ﬁndings** | | | |
| *Data analysis* | | | |
| 24. | Number of data coders | How many data coders coded the data? | - Data coding was conducted independently by two researchers |
| 25. | Description of the coding tree | Did authors provide a description of the coding tree? | - The authors provided a description of the coding tree. The manuscript details the development of a comprehensive coding framework, refined iteratively through multiple discussions. Codes were grouped into higher-order constructs, organizing success factors into broader themes referred to as segments, with internal and external factors classified accordingly. The process is described in the "Data Analysis and Synthesis" section |
| 26. | Derivation of themes | Were themes identiﬁed in advance or derived from the data? | - Themes were both identified in advance and derived from the data. The initial coding framework was informed by a systematic literature review, providing a deductive basis, while iterative discussions and thematic analysis allowed for the refinement and emergence of additional themes from the data itself, as described in the "Data Analysis and Synthesis" section |
| 27. | Software | What software, if applicable, was used to manage the data? | - For data analysis and management, ATLAS.ti was used |
| 28. | Participant checking | Did participants provide feedback on the ﬁndings? | - Success factors and associated measures were shared with participants during most interviews to validate findings and gather feedback, ensuring alignment with their experiences and insights |
| *Reporting* | | | |
| 29. | Quotations presented | Were participant quotations presented to illustrate the themes/ﬁndings? Was each quotation identiﬁed? *E.g., participant number* | - Participant quotations were presented to illustrate themes and findings in the paper. Each quotation was identified by a unique participant identifier to provide clarity and traceability while maintaining participant anonymity |
| 30. | Data and ﬁndings consistent | Was there consistency between the data presented and the ﬁndings? | - The findings were consistent with the data. Participant quotations and themes clearly supported the identified success factors and measures. |
| 31. | Clarity of major themes | Were major themes clearly presented in the ﬁndings? | - Yes, major themes were clearly presented, categorized into internal and external success factors, and supported by participant insights and illustrative quotes |
| 32. | Clarity of minor themes | Is there a description of diverse cases or discussion of minor themes? | - Yes, the paper describes diverse cases by highlighting category-specific success factors |
