# Supplementary material for: Success factors and measures for scaling patient-facing digital health technologies from leaders’ insights
Source: BMC Health Serv Res. 2025 May 1;25:632. doi: 10.1186/s12913-025-12748-z (PMC12046742; doi:10.1186/s12913-025-12748-z)
Supplement: Supplementary file 2 — Supplementary Material 2. [file 12913_2025_12748_MOESM2_ESM.docx]

**Multimedia Appendix 2: Participant selection**

Table S2: Detailed search strategy used in PitchBook to identify eligible patient-facing DHT companies for participation in the study

| **Filter** | **Search Criteria** |
| --- | --- |
| Business Statuses | Profitable; Generating revenue; Search current business status |
| Location | Europe; Search HQ Only |
| Verticals | Digital Health |
| Number of Employees | Min: 20 |
