# Supplementary material for: Success factors and measures for scaling patient-facing digital health technologies from leaders’ insights
Source: BMC Health Serv Res. 2025 May 1;25:632. doi: 10.1186/s12913-025-12748-z (PMC12046742; doi:10.1186/s12913-025-12748-z)
Supplement: Supplementary file 3 — Supplementary Material 3. [file 12913_2025_12748_MOESM3_ESM.docx]

**Multimedia Appendix 3: Interview topic guide**

Table S3: Interview topic guide outlining key questions and themes explored during semi-structured interviews with study participants

| **Topic** | **Time** | **Content** |
| --- | --- | --- |
| 1. Introduction | 5 minutes | - Welcome and purpose: introduce yourself and the study - Consent and confidentiality: confirm verbal consent to participate and to audio record the session |
| 2. Reflecting on success | 8 minutes | - What do you consider the biggest success factor that has contributed to your company's current position? This can include internal factors (e.g., team composition, business model) or external factors (e.g., stakeholder relationships, regulations) |
| 3. Measures Taken to Achieve Success Factors | 8 minutes | - What measures has your company taken to achieve [insert mentioned success factor]? |
| 4. Progress evaluation | 2 minutes | - Among these key factors, on which ones have you made the most significant progress? What specific actions or strategies led to this progress? How did these efforts impact your company’s growth or operations? |
| 5. Challenges faced | 2 minutes | - Conversely, which factors have seen the least progress? What challenges or obstacles have made progress difficult in these areas? Were there any unexpected barriers that emerged? |
| 6. Outlook | 3 minutes | - Looking ahead, do you believe the same factors will continue to drive your company's success, or do you foresee new factors becoming more crucial for growth? |
