# Supplementary material for: Success factors and measures for scaling patient-facing digital health technologies from leaders’ insights
Source: BMC Health Serv Res. 2025 May 1;25:632. doi: 10.1186/s12913-025-12748-z (PMC12046742; doi:10.1186/s12913-025-12748-z)
Supplement: Supplementary file 4 — Supplementary Material 4. [file 12913_2025_12748_MOESM4_ESM.docx]

**Multimedia Appendix 4: Codebook**

Table S4: Comprehensive codebook used for thematic analysis, including initial deductive framework and subsequent refinements based on iterative discussions

| **Category** | **Initial codes** | **Description** | **Final refined codes** |
| --- | --- | --- | --- |
| Business Models | Adaptability and flexibility to market changes and disruptions | Ability to pivot strategies, products, or services based on market dynamics, external factors, or emerging opportunities | Adaptability and flexibility to market changes and disruptions |
| Business Models | Business model consideration at an early stage | Defining and planning how the company will generate revenue and sustain profitability from the outset | Business model consideration at an early stage |
| Business Models | Business model flexibility | The ability to adjust and experiment with different business models based on market feedback and evolving conditions | Business model flexibility |
| Business Models | Competitive awareness and strategic positioning | Understanding the competitive landscape and positioning the company effectively to differentiate itself and thrive | Competitive awareness and strategic positioning |
| Business Models | Internationalization strategy | Expanding into new geographical markets, taking into account local regulations, culture, and market needs | Internationalization strategy |
| Business Models | Market positioning strategy | Strategically placing the company or product in the market to differentiate it from competitors and maximize its appeal | Market positioning strategy |
| Business Model | Sales and marketing effectiveness | Strategies and execution that drive customer acquisition, retention, and brand awareness in a cost-effective way | Sales and marketing strategy |
| Business Models | Value proposition and differentiation in the market | Clearly communicating what makes the product unique and why it offers better value compared to alternatives | Value proposition and differentiation in the market |
| Customers | Building a customer community | Fostering strong relationships with customers to create a loyal and engaged user base that supports long-term growth | Building a customer community |
| Customers | Customer feedback and satisfaction with the product/service | Regularly gathering and acting on customer insights to improve product performance and customer satisfaction | Customer feedback |
| Customers | Customer retention | Strategies and efforts focused on keeping customers engaged and preventing them from switching to competitors | Customer retention |
| Customers | Customers awareness raising | Efforts to inform and educate potential customers about the product, its benefits, and its relevance to their needs | Customers awareness raising |
| Healthcare System | Regulatory environment and policy framework | Navigating the legal and regulatory landscape to ensure compliance with evolving laws and standards | Regulatory environment and policy framework |
| Healthcare System | Improved diagnosis or care | Offering solutions that enhance medical diagnoses or improve patient care outcomes | Health impact proof and validation |
| Healthcare System | Reduction in healthcare cost | Solutions that help lower healthcare costs for providers, insurers, or patients, making care more efficient and affordable | Financial impact proof and validation |
| Product & Services | Regulatory certification | Achieving necessary approvals and certifications from regulatory bodies to legally market products, especially in healthcare | Regulatory certification |
| Product & Services | Data security, IP protection and ethics | Prioritizing data privacy, intellectual property protection, and adherence to ethical standards to build trust and ensure compliance | Data security, IP protection and ethics |
| Product & Services | Interoperability | Ensuring that products or services can integrate and communicate with other systems or devices to provide a seamless user experience | Interoperability |
| Product & Services | Regional maket size, consumer needs and behaviour | Ensuring that there is a clear demand for the product and that it addresses significant customer needs | Product-market fit |
| Product & Services | Quality and performance/brand trust | Delivering consistently high-quality products that build trust and strengthen the brand's reputation | Quality and performance |
| Partnerships | Investor backing and fit | Aligning with investors whose vision, goals, and resources support the company's long-term strategy and growth | Investor backing and fit |
| Partnerships | Synergy and alignment of sharholders' goals and objectives | Ensuring that shareholders are aligned with the company's vision and growth strategy for mutual benefit | Investor backing and fit |
| Partnerships | Alliance strategy in place | Strategic partnerships with larger firms to access new markets, share resources, or enhance credibility | Collaboration with larger organizations |
| Partnerships | Collaboration with larger organizations | Working with bigger firms to leverage their infrastructure, market reach, or expertise to scale or improve services | Collaboration with larger organizations |
| Partnerships | Key opinion leaders | Collaborating with influential figures in the industry to gain credibility and promote the product to a wider audience | Key opinion leaders |
| Employees | Diversity of skills and expertise within the team | Having a team with varied skills, backgrounds, and perspectives to drive innovation and tackle complex problems | Diversity of expertise within employees |
| Employees | Innovativeness and technical competence | Demonstrating a high level of technical expertise and creativity in developing new solutions or improving existing ones | Diversity of expertise within employees |
| Employees | Leadership experience and qualities of team members | Strong leadership that drives the team towards achieving the company's vision through experience, motivation, and decision-making | Leadership experience |
| Employees | Sense of mission | A strong, clear company purpose that motivates employees and aligns them with the broader goals of the business | Employee alignment with the company's vision |
| Employees | team alignment with the company's vision | Ensuring that all team members are aligned with and motivated by the company's overall mission and objectives | Employee alignment with the company's vision |
