# Supplementary material for: Success factors and measures for scaling patient-facing digital health technologies from leaders’ insights
Source: BMC Health Serv Res. 2025 May 1;25:632. doi: 10.1186/s12913-025-12748-z (PMC12046742; doi:10.1186/s12913-025-12748-z)
Supplement: Supplementary file 5 — Supplementary Material 5. [file 12913_2025_12748_MOESM5_ESM.docx]

**Multimedia Appendix 5: Survey Participant Demographics**

Table S5: Demographic and company characteristics of survey participants

| **Participant characteristics** | | **Participants** |
| --- | --- | --- |
| **Role, n (%)** | |  |
|  | CEO | 25 (93%) |
|  | COO | 1 (4%) |
|  | CTO | 1 (4%) |
| **DHT category, n (%)** | |  |
|  | Care Support | 4 (15%) |
|  | Digital Diagnostics | 5 (19%) |
|  | Digital Therapeutics | 5 (19%) |
|  | Health & Wellness | 6 (22%) |
|  | Patient Monitoring | 7 (26%) |
| **Number of Employees, n (%)** | |  |
|  | 20-50 | 14 (52%) |
|  | 50-100 | 5 (19%) |
|  | 100-200 | 3 (11%) |
|  | 200+ | 5 (19%) |
|  |  |  |
