# Supplementary material for: Success factors and measures for scaling patient-facing digital health technologies from leaders’ insights
Source: BMC Health Serv Res. 2025 May 1;25:632. doi: 10.1186/s12913-025-12748-z (PMC12046742; doi:10.1186/s12913-025-12748-z)
Supplement: Supplementary file 6 — Supplementary Material 6. [file 12913_2025_12748_MOESM6_ESM.docx]

**Multimedia Appendix 6: Survey Questions**

Table S6: Follow-up survey questions and response options for validating success factors and implementation measures

| **Questions** | | **Response options** |
| --- | --- | --- |
| What are the most important factors for scaling digital health companies? | | - Business model flexibility - Internationalization strategy - Market positioning strategy - Sales and marketing strategy - Product-market fit - Regulatory certification - Quality and performance - Leadership experience - Diversity of expertise within employees - Employee alignment with the company's vision - Health impact proof and validation - Regulatory environment and policy framework - Financial impact proof and validation - Customer feedback - Customers awareness raising - Regional maket size and customer behaviour - Collaboration with larger organizations - Investor backing and fit |
| *The following questions appear only if the relevant success factor has been selected* | | |
|  | What are measures to achieve **business model flexibility**? | - Rapid experimentation and market adaptation - Tailoring to diverse customer needs - Pivoting quickly from failing models - Other (enter text) |
|  | What are the important measures to achieve a successful **internationalization strategy**? | - Strategic market selection and timing - Adapting to local market conditions - Leveraging partnerships for market entry - Other (enter text) |
|  | What are important measures for a successful **market positioning strategy**? | - Start in non-medical or less regulated markets - Strategic regulatory management - Clear communication of product capabilities - Other (enter text) |
|  | What are important measures for a successful **sales and marketing strategy**? | - Direct-to-consumer awareness and trust building - Leveraging partnerships for scaled sales - Data-driven customer management and optimization - Other (enter text) |
|  | What are important measures to achieve **product-market fit**? | - Clear unmet need identification - Extensive market research and validation - Targeting high-opportunity markets - Other (enter text) |
|  | What are the important capabilities to achieve **regulatory certification**? | - Strategic regulatory planning - Building clinical evidence and meeting quality standards - Ensuring compliance with data privacy regulations - Other (enter text) |
|  | What are important measures to achieve high **quality and performance** of product/service? | - Building trust through authenticity and research - Providing unique solutions tailored to customer needs - Self-regulation for continuous improvement - Other (enter text) |
|  | What are important **leadership** capabilities? | - Industry-specific leadership experience - Cohesion and trust among leadership - Determination and ability to execute - Other (enter text) |
|  | What are key to achieve **diversity of expertise** within employees? | - Balancing expertise across disciplines - Ensuring a wide range of capabilities - Assembling the team based on problem-solving needs - Other (enter text) |
|  | What are key measures to achieve **employee alignment with the company's vision**? | - Careful selection of passionate team members - Transition from hero culture to team culture - Other (enter text) |
|  | What are key measures to achieve **health impact proof and validation**? | - Building clinical evidence and health economics - Leveraging clinical trials for trust and validation - Demonstrating outcomes over time - Other (enter text) |
|  | What are key measures to align with the **regulatory environment and policy framework**? | - Leveraging reimbursement models for market access - Balancing regulatory compliance with scalability - Staying informed and proactive on regulatory changes - Other (enter text) |
|  | What are the important measures to achieve **financial impact proof and validation**? | - Demonstrate cost savings with data-backed case studies - Translate clinical benefits into financial impact - Other (enter text) |
|  | What are the key requirements to collect **customer feedback**? | - Regular engagement with customers - Validating product ideas early through customer input - Prioritzing customer support - Other (enter text) |
|  | What are key measures to ensure proper **customer awareness raising**? | - Leverage key opinion leaders and ambassadors for credibility - Educate the market through multi-channel campaigns - Address resistance to new technologies - Other (enter text) |
|  | What are key measures to find the best **market size and customer segment**? | - Prioritize markets with high demand and receptive attitudes towards innovation - Adapt go-to-market strategies to regional customer behavior - Other (enter text) |
|  | What are important measures to achieve successful **collaboration with larger organizations**? | - Leverage intellectual property to align with larger partners - Establish paid partnerships for long-term commitment - Other (enter text) |
|  | What are key measures to achieve **investor backing and fit**? | - Choosing investors who offer "Smart Money" - Ensuring investor and founders vision align - Leveraging network for funding" - Other (enter text) |
